# Initiation and continued use of oral pre-exposure prophylaxis among pregnant and postpartum women in South Africa (PrEP-PP): a demonstration cohort study

**DOI:** 10.1016/S2352-3018(24)00240-6

**Published:** 2024-11

**Authors:** Dvora Leah Joseph Davey, Rufaro Mvududu, Nyiko Mashele, Kalisha Bheemraj, Nehaa Khadka, Leigh F Johnson, Sarah Schoetz Dean, Pamina Gorbach, Linda-Gail Bekker, Thomas J Coates, Landon Myer

**Affiliations:** Division of Infectious Diseases, David Geffen School of Medicine, University of California Los Angeles, Los Angeles, CA, USA; Division of Epidemiology and Biostatistics, School of Public Health, University of Cape Town, Cape Town, South Africa; Department of Epidemiology, Fielding School of Public Health, University of California Los Angeles, Los Angeles, CA, USA; Division of Epidemiology and Biostatistics, School of Public Health, University of Cape Town, Cape Town, South Africa; Division of Epidemiology and Biostatistics, School of Public Health, University of Cape Town, Cape Town, South Africa; Division of Epidemiology and Biostatistics, School of Public Health, University of Cape Town, Cape Town, South Africa; Division of Infectious Diseases, David Geffen School of Medicine, University of California Los Angeles, Los Angeles, CA, USA; Centre for Infectious Disease Epidemiology and Research, University of Cape Town, Cape Town, South Africa; Division of Infectious Diseases, David Geffen School of Medicine, University of California Los Angeles, Los Angeles, CA, USA; Department of Epidemiology, Fielding School of Public Health, University of California Los Angeles, Los Angeles, CA, USA; Division of Infectious Diseases, David Geffen School of Medicine, University of California Los Angeles, Los Angeles, CA, USA; The Desmond Tutu HIV Centre, University of Cape Town, Cape Town, South Africa; Division of Infectious Diseases, David Geffen School of Medicine, University of California Los Angeles, Los Angeles, CA, USA; Division of Epidemiology and Biostatistics, School of Public Health, University of Cape Town, Cape Town, South Africa

## Abstract

**Background:**

When used effectively, oral pre-exposure prophylaxis (PrEP; tenofovir disoproxil fumarate and emtricitabine) prevents maternal HIV acquisition and reduces the risk of vertical transmission. Our study aimed to better understand PrEP initiation, continued use, and adherence in pregnant and postpartum women.

**Methods:**

The PrEP in Pregnancy and Postpartum (PrEP-PP) study is a demonstration cohort study that enrolled pregnant women aged 16 years and older without HIV attending their first antenatal care visit in Cape Town, South Africa, between Aug 29, 2019, and Oct 10, 2021. Eligible, consenting women were followed up quarterly up to 12 months postpartum with regular HIV testing and offer of PrEP with ongoing adherence counselling. The primary outcome was distribution of women across the PrEP cascade (ie, initiation and continuation up to 12 months postpartum) with crude and adjusted hazard ratios (HRs). We also report on HIV incidence by pregnancy and postpartum status.

**Findings:**

Overall, 1195 pregnant women were recruited and followed up (median age 26 years, IQR 23–31; median gestational age 21 weeks, IQR 15–31); 1009 (84·4%) started PrEP at enrolment. Among women who initiated PrEP at enrolment, 668 (67·5%) of 990 continued PrEP at the 1-month follow-up, 485 (49·9%) of 972 continued at 3 months, 392 (39·4%) of 994 at 6 months, and 275 (27·4%) of 1005 at 12 months. Of 186 women who did not accept PrEP at enrolment, 70 (37·6%) of 186 subsequently initiated PrEP. Overall, 200 (18·6%) of 1076 women continued PrEP at 12 months postpartum. Of 186 women who did not initiate PrEP at baseline, 70 (37·6%) subsequently initiated PrEP during the study. Factors associated with PrEP discontinuation up to 12 months postpartum included being married or cohabiting (adjusted HR 1·32, 95% CI 1·16–1·50), condomless sex since last visit (1·43, 1·23–1·65), reporting intimate partner violence (2·03, 1·59–2·59), or depression in the past 12 months (1·53, 1·14–2·05). Overall, 16 women seroconverted over 1673·8 woman-years (HIV incidence rate 0·96 per 100 woman-years, 95% CI 0·49–1·42); 14 discontinued PrEP use and two never initiated PrEP. HIV incidence was 0·28 per 100 woman-years during pregnancy (95% CI 0·22–0·33), and the incidence rate ratio was 1·77 per 100 woman-years (0·53–5·90) 0–6 months postpartum and 2·19 per 100 woman-years (0·61–7·83) 6–12 months postpartum compared with pregnant women.

**Interpretation:**

There is an urgent need for the integration of PrEP into antenatal and postnatal care and interventions that address barriers to continued use, including targeted counselling during pregnancy and postpartum to reduce PrEP discontinuation.

**Funding:**

National Institute of Mental Health and Fogarty International, US National Institutes of Health.

## Introduction

Over half of global HIV infections occur among cisgender women, with notably high HIV incidence among women who are pregnant and postpartum.^1,2^ Increased risk of HIV acquisition during gestation due to biological and behavioural changes coupled with the potential for vertical transmission make HIV prevention among pregnant and lactating people (PLP) a unique and crucial global health priority.^2,3^

In the past 5 years, research efforts have prioritised understanding the effectiveness and acceptability of biomedical HIV prevention among PLP, including oral tenofovir disoproxil fumarate and emtricitabine pre-exposure prophylaxis (PrEP).^4,5^ These findings have provided valuable insights into the safety and efficacy of PrEP,^6–8^ informing both WHO recommendations^9^ and subsequent 2021 South African National Guidelines to include offering PrEP to PLP.^10^ However there are few data on how to provide and optimise PrEP use among PLP.^11^ The use of oral PrEP is increasing among PLP, with notable successes in Kenya, South Africa, and Zimbabwe.^12,13^ Despite growing availability and uptake, PrEP persistence and effective adherence (daily use before and during periods of sexual activity) are major challenges for effective use among pregnant women, and especially among breastfeeding women.^11,14,15^ As these elements of PrEP use are integral to prevention effectiveness, future research exploring the suitable delivery of PrEP and how to best optimise PrEP use among PLP is urgently needed.^11,16^

Previous studies have highlighted existing physical and social barriers contributing to suboptimal PrEP use among cisgender women, such as difficulties in clinical access, daily pill burden, side-effects, insufficient interpersonal support, and anticipated PrEP-related stigmas.^4,12,17^ In similar studies in Kenya and South Africa, PrEP discontinuation frequently occurred within the early months of use and was attributed to changes in HIV risk perception and side-effects experienced, which can worsen in early pregnancy.^4,12^ Another crucial point of PrEP discontinuation has been observed during the perinatal period and transition from antenatal to postnatal care, as women can perceive the infant risk of HIV acquisition to be significantly diminished following birth or have difficulty adhering to a daily pill regimen due to the demands of early parenthood.^18^ We aimed to evaluate PrEP initiation, continuation, and correlations of continued PrEP use among a cohort of South African PLP without HIV in antenatal care.

## Methods

### Study design

The PrEP in pregnancy and postpartum (PrEP-PP) study is a demonstration cohort study conducted in Cape Town, South Africa. PrEP-PP started before PrEP was part of the standard of care for pregnant women (South African guidelines were updated in 2022 to integrate PrEP for PLP). Study staff were trained to implement all study visits, including data collection, counselling, laboratory analysis, and PrEP prescriptions. The study method has been described in detail previously.^12^

The Human Research Ethics Committee at the University of Cape Town (#297/2018) and the University of California, Los Angeles Institutional Review Board (IRB#18-001622) approved this study. All study participants were reimbursed for their time (ZAR 150 per visit) in grocery vouchers. The study protocol is available online at ClinicalTrials.gov (NCT03902418).

### Participants

We recruited pregnant women without HIV at the first antenatal care visit at a single clinic between Aug 29, 2019, and Oct 10, 2021. The government-operated primary care facility was purposively selected for recruitment on the basis of the diversity of the surrounding township with a high antenatal HIV prevalence. Pregnant women were contacted about the study after a health talk in the clinic about HIV treatment and prevention to prevent vertical transmission. We recruited and enrolled eligible pregnant women who were aged 16 years or older, not living with HIV (with a 4th generation rapid HIV antigen and antibody test), hepatitis B surface antigen negative (with a rapid hepatitis B surface antigen test), intending to give birth in Cape Town, and had no medical contraindications to PrEP. Women were eligible to enrol regardless of estimated gestation weeks at first antenatal care, including those with previous or current PrEP use. All participants provided written informed consent in English or their local language (ie, isiXhosa). Unassisted consent was obtained from pregnant adolescents aged 16–17 years. After providing consent, the women were followed up quarterly through delivery until 12 months postpartum. HIV counselling and testing were provided, followed by HIV prevention counselling (including safety and effectiveness of PrEP), and PrEP support at each study visit.

### Procedures

Trained study staff administered a baseline survey collecting participants’ demographic information (sex was self-reported as sex at birth), clinical characteristics, and sexual behaviours from the past 12 months and 3 months. Participants provided self-collected vaginal swabs at enrolment for near patient diagnosis of sexually transmitted infections (STIs; *Chlamydia trachomatis*, *Neisseria gonorrhoeae*, and *Trichomonas vaginalis*) with GeneXpert (Cepheid, Sunnyvale, CA, USA).

Those diagnosed with STIs were provided treatment per South African guidelines. Trained study counsellors provided counselling at baseline and quarterly on HIV risk during pregnancy and postpartum, ways to prevent HIV (eg, condoms and partner HIV testing), and the benefits and risks of taking PrEP to prevent HIV. Participants were asked if they would like to initiate PrEP, with the clarification that the use of PrEP at any time would not affect their study participation. For women on PrEP, adherence counselling included how best to remember to take the daily pill (eg, set a mobile phone alarm), and how best to talk about PrEP with family and partners with the provision of a PrEP brochure to support disclosure. The study nurses drew blood from those starting PrEP to confirm baseline creatinine concentrations in line with the national PrEP guidelines.^10^ Participants who initiated PrEP were provided a month’s supply of tenofovir disoproxil fumarate and emtricitabine dosage, followed by quarterly supplies thereafter.

Quarterly study visits aligned with antenatal and postpartum or well-baby immunisation visits. Study interviewers collected information following surveys that included updated data on sexual behaviours, relationship status, and PrEP adherence. Participants were asked if they wanted to continue, start, or restart PrEP at each study visit. During follow-up, nurses collected dried blood spots from a subset of women who reported taking PrEP in the past 30 days for analysis of measurable concentrations of tenofovir diphosphate as a measurement of adherence.^19^

Exposures in the analysis included age, relationship status, education, and ongoing data on emotional, physical, or sexual intimate partner violence, drug and alcohol use in the past 12 months (Drug and Alcohol Use Disorders Identification Test), and depression (the Edinburgh Postnatal Depression Scale [EPDS]^20^). We collected additional data quarterly on sexual behaviours, including number of sexual encounters in the past month, condom use at last sexual encounter and in past 3 months, number of sexual partners, and partner HIV status. We also recorded the STI diagnosis and treatment at baseline. We used peer counsellors to interview participants to reduce reporting biases. We conducted sensitivity analyses to compare exposures and outcomes to evaluate biases.

### Outcomes

The primary outcome was the distribution of women across the PrEP cascade, including initiation (ever received PrEP prescription during study) and continuation on PrEP (received PrEP prescription in review prescription records), up to 12 months postpartum via crude and adjusted hazard ratios (HRs). Secondary outcomes included HIV incidence in pregnancy and postpartum and adherence (ie, proportion of women with objective adherence at 6 months measured with erythrocyte intracellular tenofovir diphosphate concentrations). Adherence was measured with liquid chromatography and mass-spectrometry from dried whole blood spots, providing a measure of cumulative PrEP adherence over several weeks.^19^ We defined objective PrEP adherence as any tenofovir diphosphate detected in collected dried blood spots at the quarterly study visits among those who initiated PrEP at baseline and those whose dried blood spots were collected and analysed. We analysed a random selection of 186 dried blood spots samples at the 6-month follow-up in participants who reported PrEP use in the past month. Analysis used previously published adherence thresholds of tenofovir diphosphate (>1000 fmol/punch for taking seven doses a week, 400–999 fmol/punch for taking two to six doses a week, and <400 fmol/punch for taking less than two doses a week).^19^

### Statistical analysis

A target enrolment of 1200 pregnant women intended to provide 80% power to identify an HR of 2·0 or greater for evaluating differences in age (<25 years *vs* ≥25 years) on PrEP continuation through the postpartum period. PrEP continuation and discontinuation were reported among participants who initiated PrEP at baseline or at any time during the study (the primary endpoint was 12 months postpartum, thus we measured initiation through to 9 months postpartum, the second to last visit, to evaluate initiation). Discontinuation of PrEP at 12 months postpartum was defined as participants who self-reported no PrEP use in the past 3 months, did not receive a PrEP prescription for more than 3 months, missed the 12-month postpartum visit, or were censored before the 12-month postpartum visit due to seroconversion, pregnancy loss, or infant death.

Crude and adjusted HRs from the Cox proportional hazard models accounted for the total time on PrEP until first discontinuation (no prescription for >3 months) up to 12 months postpartum or until the censorship event, adjusted for a priori confounders, including maternal age (continuous), gestational age (continuous), and educational level (categorical) at baseline. We used directed acyclic graphs to find out which a priori confounders should be included in the models. To assess assumptions in proportional hazard models, Schoenfeld residuals were calculated and visually inspected for each predictor variable plotted over time. Kaplan–Meier curves present the occurrence of PrEP discontinuation and show the probability of PrEP continuation over time, stratified by age and pregnancy versus postpartum status. Multiple Cox proportional hazard models were fitted to isolate the effect of each characteristic while controlling for a priori confounders to avoid potential interactions in a more complex model. We applied a Bonferroni correction to ensure the reliability of the findings, with the type 1 error rate set at 0·0042. Since the significance of all variables remained unchanged, we proceeded with the Cox proportional hazard models and reported the corresponding p values.

Objectively measured tenofovir diphosphate at 6 months compared participants with any tenofovir present (ranging from low adherence of <2 doses a week to high adherence of 7 doses a week) versus those with no tenofovir present (with a tenofovir result below the limit of quantification) with logistic regression adjusted for a priori confounders. We ran a sensitivity analysis using log-binomial models, which did not change any of the estimates in the logistic regression models. HIV incidence was based on the time in study and the number of seroconversions during the study period and reported as a rate per 100 woman-years. The date of seroconversion is reported as the date of HIV testing. Subgroup analyses were conducted to assess HIV incidence during pregnancy and early and late postpartum periods and among those who continued, discontinued, or never started PrEP. Comparisons of Poisson rates and generalised estimating equations were used to compare incidence rates within these subgroups. Statistical tests were two-sided with an α of 0·05, except those subject to Bonferroni correction using an α of 0·0042. All analyses were performed in RStudio version 4.3.1.

### Role of the funding source

The funders of the study had no role in study design, data collection, data analysis, data interpretation, or writing of the report.

## Results

We assessed eligibility among 2554 pregnant women between August, 2019, and October, 2021. Overall, 1359 (53·2%) of 2554 women were excluded due to study exclusion criteria (738 [54·3%] of 1359 women), declining participation in the study (407 [29·9%] of 1359 women), not returning for the start of the study (108 [7·9%] of 1359 women), or participation in another HIV study (106 [7·8%] of 1359 women). We enrolled 1195 pregnant women in the PrEP-PP cohort study at their first antenatal care visit (with no limit on gestational age at enrolment), and follow-up continued up to Feb 9, 2023 (appendix 2 p 2).

The median age of the participants was 26 years (IQR 23–31). Of 1195 women, 457 (38·2%) were younger than 25 years, 450 (37·7%) were either married or cohabiting with their partner, 1162 (97·2%) reported being sexually active during pregnancy, 864 (72·3%) reported never or rarely using a condom during sex in the past 3 months, and 373 (31·2%) were diagnosed with an STI at baseline. 147 (12·3%) reported intimate partner violence (sexual, emotional, or physical) in the past 12 months and 332 (28·1%) of 1181 either had a current partner living with HIV (20 [1·7%]) or had partners of unknown serostatus (312 [26·4%]; table 1).

**Figure 1:**
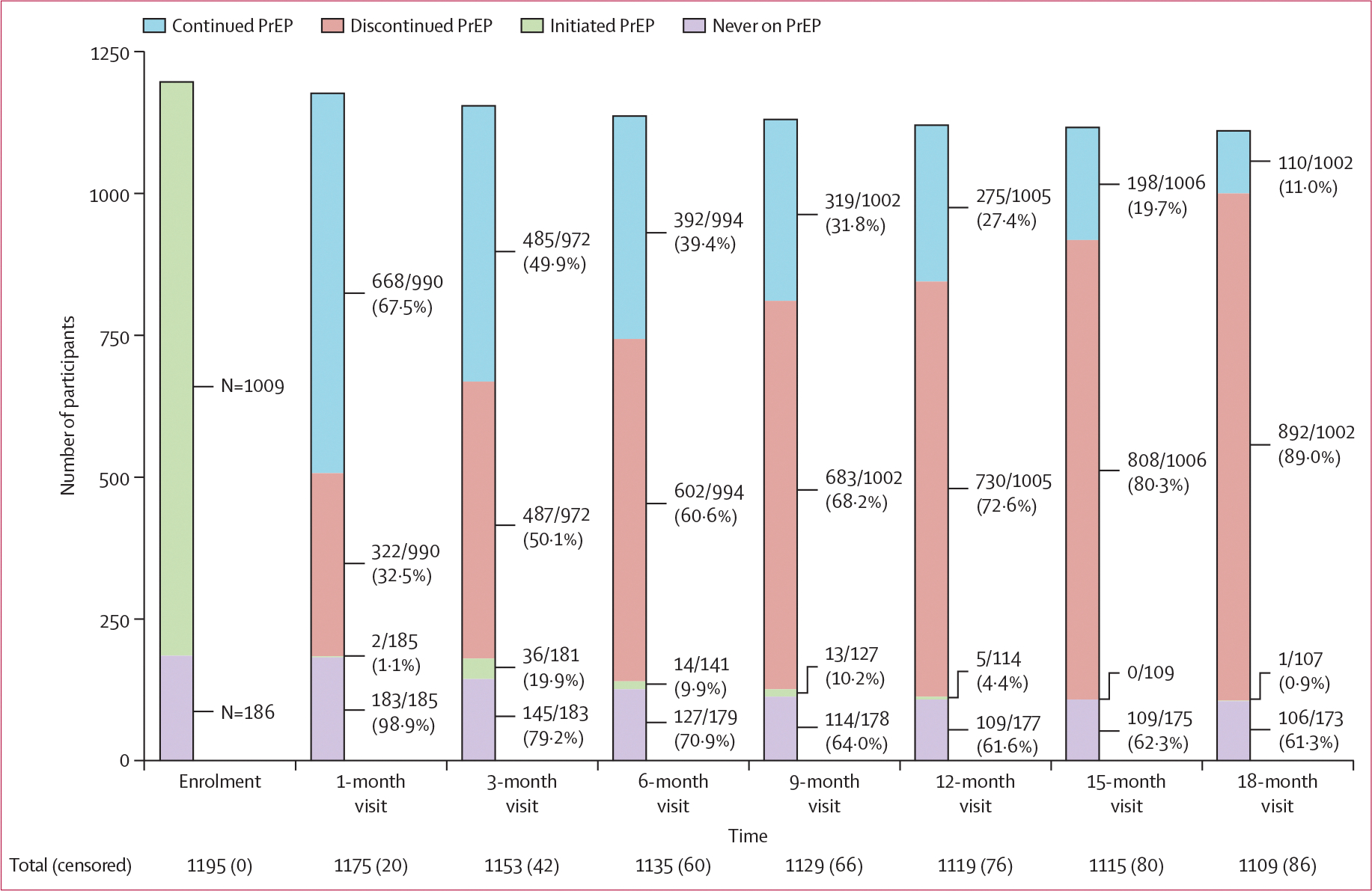
Cascade of oral PrEP use in cohort of pregnant and postpartum women in PrEP-PP study, Cape Town, South Africa (2018–23) Continued PrEP: participants who started PrEP at prior visit and returned for refill prescription within the time frame. Discontinued PrEP: participants who started PrEP at prior visit but did not return for subsequent visit to receive prescription or did not want to continue PrEP at this visit (refused repeat prescription). Initiated PrEP: participants who started PrEP at that visit (received first PrEP prescription in study), for those who were on PrEP before and restarted, were considered as continuation (not initiation). Never on PrEP: participants who never received a PrEP prescription. Participants were censored at each study visit due to pregnancy loss, infant death, or seroconversion. PrEP=pre-exposure prophylaxis.

1009 (84·4%) of 1195 pregnant women initiated PrEP at baseline. Of the women who initiated PrEP, 668 (67·5%) of 990 continued at the 1-month follow-up and 322 (32·5%) discontinued PrEP or missed the study visit. At 3 months, 485 (49·9%) of 972 women continued PrEP and 487 (50·1%) discontinued or missed the study visit, and 36 (19·9%) of 181 women who did not start PrEP at baseline initiated PrEP. Attrition in PrEP continuation continued as the study progressed, with 392 (39·4%) of 994 women continuing PrEP at 6 months, 319 (31·8%) of 1002 continuing at 9 months, 275 (27·4%) of 1005 continuing at 12 months, 198 (19·7%) of 1006 continuing at 15 months, and 110 (11·0%) of 1002 continuing at 18 months (figure 1). Overall, 285 (32·0%) of 892 of women attended the study visits and elected to discontinue PrEP. 200 (18·6%) of 1076 of women continued PrEP at 12 months postpartum. Of 186 women who did not initiate PrEP at baseline, 70 (37·6%) subsequently initiated PrEP during the study (figure 1). Common reasons for discontinuation included side-effects, not having a sex partner, or not being sexually active. Among 892 women who discontinued taking PrEP throughout the study, 246 (27·6%) opted to restart PrEP at a follow-up visit. At the end of the study, 106 (8·9%) of 1195 women had never started PrEP. 86 (7·2%) of 1195 women were censored from the study due to pregnancy loss, infant death, or seroconversion (appendix 2 p 2). Gestational age and postpartum time in each study visit is reported in appendix 2 (p 2).

**Figure 2:**
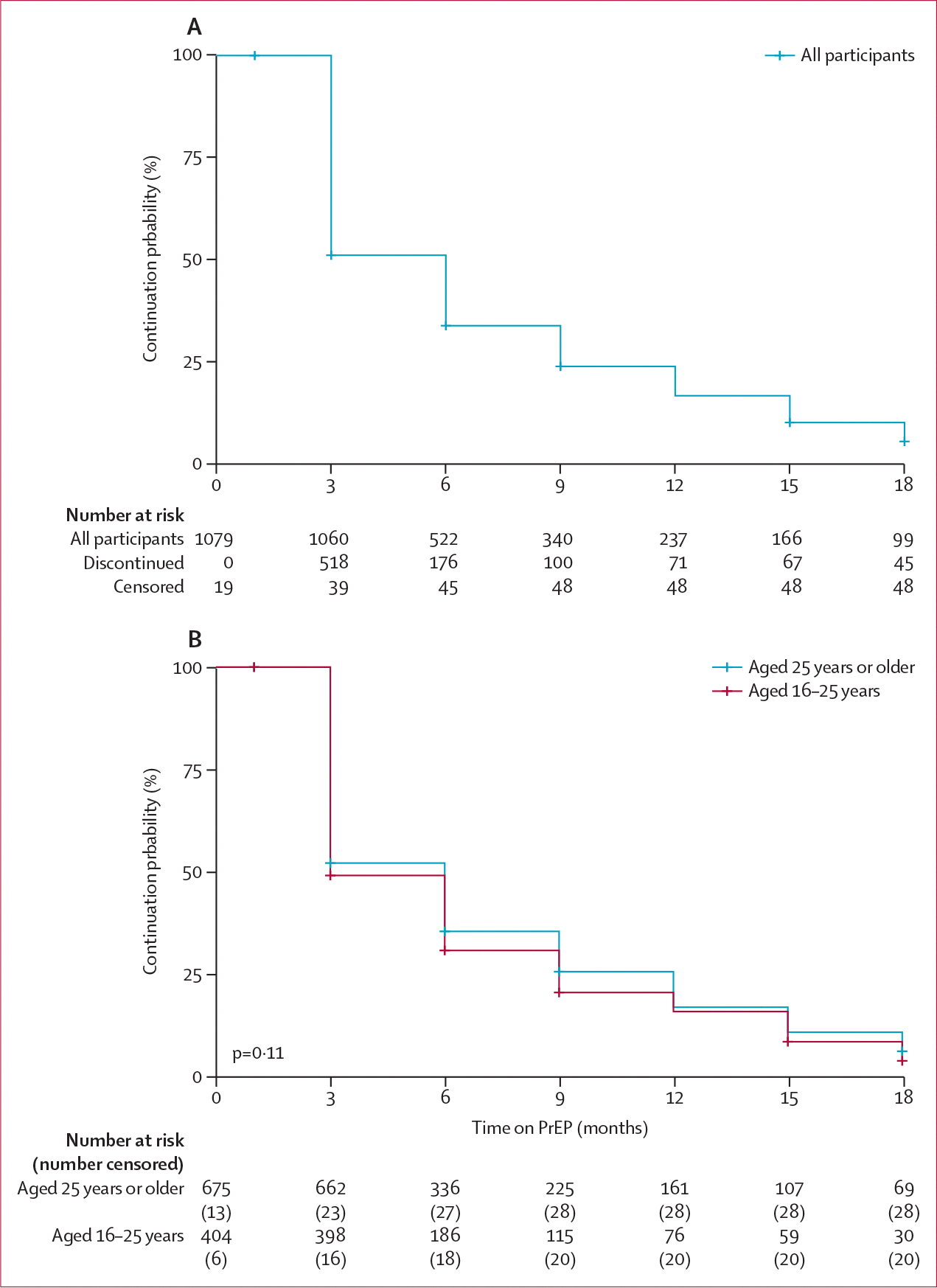
Kaplan–Meier curves of first PrEP discontinuation in PrEP-PP study in Cape Town, South Africa (2018–23) Among all participants who were ever on PrEP before study end (n=1079; A) and stratified by age across time on PrEP (16–25 years *vs* ≥25 years). Discontinued PrEP defined as participants who started PrEP at previous visit but did not return for subsequent visit to receive prescription or did not want to continue PrEP at this visit (refused repeat prescription). Participants were censored at each study visit due to pregnancy loss, infant death, or seroconversion. PrEP=pre-exposure prophylaxis.

The largest drop in PrEP continuation was in the first month when 322 (31·9%) of 1010 women either discontinued PrEP or were censored due to pregnancy loss. We also stratify by age in the Kaplan–Meier curve, which showed that women aged 16–24 years discontinued PrEP use at a quicker rate than older women, although the difference between age groups was not statistically significant. Overall, 892 (89·5%) of 1031 of women who started PrEP discontinued by 12 months postpartum (figure 2). During their pregnancy period, 525 (52·0%) of 1009 women who initiated PrEP at baseline had discontinued PrEP for the first time.

We evaluated our primary outcome of PrEP continuation at 12 months postpartum (table 2). Correlations of PrEP discontinuation at 12 months postpartum included later gestational age at PrEP initiation (≥20 weeks gestational age *vs* <20 weeks gestational age; adjusted HR 1·31, 95% CI 1·15–1·49), being married or cohabiting versus unmarried or not cohabiting (1·32, 1·16–1·50), having a partner of unknown serostatus (3·03, 2·44–3·77), sexual activity in past 3 months versus none (1·47, 1·25–1·74), reported condoms never or rarely used in past 3 months (1·43, 1·23–1·65), reported intimate partner violence in past 3 months (2·03; 1·59–2·59), or reported depression (EPDS threshold ≥11 *vs* <11; 1·53, 95% CI 1·14–2·05). Any alcohol use in past 3 months was associated with lower risk of PrEP discontinuation (adjusted HR 0·71, 95% CI 0·56–0·89).

Among a subset of 186 women who reported taking PrEP in the past month at the 6-month follow-up, 86 (46·2%) had tenofovir diphosphate present in their dried blood spots, of whom 41 (47·7%) had low concentrations indicating less than two doses a week, 40 (46·5%) had concentrations equivalent to an estimated two to six doses a week, and five (5·8%) had high concentrations indicating approximately seven doses a week. Younger age (aged 16–24 years *vs* aged ≥25 years, adjusted odds ratio [OR] 2·33, 95% CI 1·19–4·69) and secondary school or tertiary education compared with primary school (adjusted OR 1·81, 95% CI 0·99–3·33) were associated with increased odds of not having tenofovir diphosphate in dried blood spots, a proxy for PrEP adherence in the past month (appendix 2 pp 1, 3).

Overall, 16 women seroconverted over 1673·8 woman-years of follow-up, with an HIV incidence rate of 0·96 per 100 woman-years (95% CI 0·49–1·42). During pregnancy, one woman seroconverted (incidence rate 0·28 per 100 woman-years, 95% CI 0·22–0·33). The postpartum HIV incidence rate ratio was 1·77 per 100 woman-years less than 6 months postpartum (95% CI 0·53–5·90) and 2·19 per 100 woman-years 6–12 months postpartum (0·61–7·83) compared with pregnancy incidence rates. In women who never started PrEP, the HIV incidence rate was 1·28 per 100 woman-years (95% CI 0·48–3·03; n=2), and 0·92 per 100 woman-years (0·49–1·52; n=14) in women who discontinued PrEP before seroconversion (table 3 and appendix 2 p 2).

## Discussion

These data provide crucial insights into the delivery of oral PrEP to public sector antenatal and postnatal care in a setting with a high HIV prevalence. Despite the high rate of initiation of oral PrEP (84%) among women at high risk of HIV acquisition at the first antenatal care visit, a third of pregnant women who started PrEP discontinued after a month, and persistence and adherence remained low during follow-up, pointing to the challenges in ensuring effective PrEP use in this population. Women who were married or cohabiting with their partner or reported intimate partner violence or condomless sex in the past year were more likely to discontinue PrEP. This study is one of the first studies integrating PrEP into a South African public clinic^21^ and it contributes toward understanding PrEP use and continuation, barriers and facilitators, and HIV incidence among PLP over time to inform appropriate future interventions, ultimately improving PrEP uptake and persistence across the pregnancy, peripartum, and postpartum periods. Our study was implemented before PrEP was routinely provided for PLP in South Africa, which might have presented a barrier and affected continuation of PrEP.

Given the established knowledge of the increased risk of HIV acquisition in pregnant and postpartum women, further insights into the use of PrEP during these stages are urgently needed. Thus far, data on PrEP use among PLP vary; a clinical trial published in 2023 spanning 20 maternal child health clinics in Kenya observed low initial uptake of PrEP (14%) but higher uninterrupted continuation among mothers 9 months postpartum (58%).^17^ Our study revealed high initial uptake of PrEP in antenatal care, which might be due to elevated HIV prevalence in the community compared with Kenya. Our study might overestimate true interest in PrEP initiation, as although PrEP use was optional, 30% of women approached declined participation in the study. Participants who decided to enrol in the study might have high-risk behaviours or partners living with HIV (or unknown serostatus), compared with those who opted out of participating. Participants initiating PrEP during pregnancy in South Africa have described not only conventional barriers to continuation, such as pill burden, stigma, and logistical challenges, but also unfamiliar hurdles unique to pregnancy and childbirth, such as important shifts in lifestyle, physiology, and sexual behaviours.^22,23^ In PrEP-PP, a third of women who started PrEP but returned for study visits stopped PrEP during the study, mostly due to reporting side-effects (especially nausea and vomiting) and changes in sexual activity (no sex partner) or stigma around taking PrEP (anticipated or experienced). Interventions that offer strategies to improve PrEP continuation, including how best to address side-effects in pregnancy and when to resume taking PrEP before resuming sexual activity postpartum, could benefit PLP.

Our previous analysis of early PrEP discontinuation showed that postpartum women were more likely to discontinue PrEP use than pregnant women, and tenofovir diphosphate concentrations were lower in pregnancy than in postpartum^14^ (stratified adherence comparing pregnancy *vs* postpartum^19^), highlighting the need for continued PrEP adherence counselling and support to restart PrEP before sexual activity resumes in the postpartum period.^12^ Furthermore, integrating PrEP services into clinical settings in which postpartum women are consistently present, such as child wellness or family planning visits, could diminish hurdles in PrEP continuation among those who are no longer engaged in antenatal care.^24^

We identified important factors associated with PrEP discontinuation that can be used to inform additional adherence support or identify priority populations for longer acting PrEP methods, including injectable and ring PrEP. In women who struggle with daily oral PrEP use, long-acting injectables^25^ or vaginal rings^26^ could help alleviate barriers. For women who have difficulty attending regular clinic visits, differentiated delivery of PrEP outside of the clinic (eg, in community or private pharmacy settings) could improve its effective use over time, especially in the postpartum period. Alternatively, counselling on the past 72 h adherence via urine testing for tenofovir could be another cost-effective, simple intervention to improve adherence.^27^ Furthermore, women who were younger, primigravid, married or cohabiting with their partner, reported condomless sex, or had a partner with unknown serostatus were more likely to discontinue PrEP. Interventions including continued counselling to improve the disclosure of PrEP status to partners and family and providing facility-based or at-home self-diagnostic HIV testing to partners could improve risk perception and target those who need PrEP most, including those in serodiscordant relationships.^27^

Notably, a fifth of women who did not start PrEP at baseline started PrEP at later visits during the postpartum period. The early adoption period of PrEP might be challenging for many soon-to-be mothers, potentially because of factors such as anticipated or experienced negative physical side-effects of PrEP overlapping with those of pregnancy, financial or logistical strains, or the preoccupation of preparing for imminent childbirth.^28^ In postpartum women who continued PrEP at 6 months, less than half had concentrations of tenofovir diphosphate in blood spots, and of those women, half had low concentrations (<2 doses a week), identifying important barriers to daily PrEP adherence in postpartum women. Furthermore, the incidence of HIV was high among postpartum women who discontinued PrEP. These observations emphasise the necessity of offering PrEP throughout postpartum and breastfeeding periods, not just in early pregnancy, along with consistent counselling and messaging on the importance of its effective use following childbirth.

As HIV prevention technologies expand to include novel forms of PrEP and differentiated delivery of PrEP, gathering insight from PLP into HIV prevention modality, frequency, and delivery preferences is crucial for enhancing PrEP continuity during this pivotal transition period. A 2023 study of PLP on oral PrEP from PrEP-PP and a Kenya-based study showed high theoretical preference for injectable PrEP over oral PrEP (75% of participants).^29^ There is great value in ensuring that PLP have access to a wide variety of PrEP modalities, in tandem with development of tailored postpartum programming and delivery options that highlight the flexibility of PrEP, which could further support the effective use of PrEP among PLP.

These HIV incidence data suggest that PrEP is highly effective when used consistently, in line with modelling in the past year showing 90% protection when women took some product.^30^ Most HIV acquisition occurred postpartum, with overall postpartum HIV incidence most elevated 6–12 months postpartum (when half of women were still breastfeeding), especially among women who had started PrEP but discontinued use. Furthermore, HIV incidence was low during pregnancy when most women continued taking PrEP and had higher concentrations of tenofovir diphosphate (proxy for recent adherence).^14^ These findings highlight the urgent need for programmes that promote PrEP continuation and adherence in PLP, particularly in the period following childbirth, through interventions such as offering long-acting PrEP methods and differentiated delivery of PrEP, which might particularly benefit women no longer accessing antenatal or postpartum care.

The strengths of this study included the longitudinal study design incorporating measures of HIV acquisition, allowing for the ability to capture and analyse granular data regarding not only PrEP continuation versus discontinuation, but also missed clinic visits and participants re-starting PrEP throughout the period of observation. Despite its strengths, the generalisability of the findings might be limited, as this study population comprised only those attending a single antenatal care setting in one city. However, our use of study personnel for PrEP delivery contributed to increased internal validity and data completion. Additionally, the results pose the potential for outcome misclassification, as PrEP continuation in this analysis was based on prescription collection and not on objective measurements of PrEP adherence, and therefore is subject to social desirability bias, especially due to data collection and repeat prescriptions provided by study staff. Furthermore, tenofovir diphosphate was only measured in those who reported using PrEP in the past month, which might overestimate true adherence.

These findings provide evidence that PrEP use during pregnancy and breastfeeding is non-uniform and complex, requiring multidimensional interventions that are tailored to the experiences of women during these periods. PrEP programming should consider additional targets that focus on improving PrEP continuation and adherence among young PLP. Additionally, given the high incidence of HIV in the postpartum period among those who discontinued PrEP observed in this cohort, PLP (and postpartum women specifically) might benefit from enhanced counselling, partner HIV testing, long-acting methods, and differentiated delivery of PrEP if women are no longer accessing antenatal or postnatal care services, but are still at elevated risk of HIV acquisition and vertical transmission. As HIV biomedical prevention is swiftly advancing, the inclusion of PLP in both research and interventions is essential to ending HIV.

## Supplementary Material

Supplemental tables

French abstract

## Figures and Tables

**Table 1:** Baseline sociodemographic, clinical, and behavioural characteristics of pregnant women offered pre-exposure prophylaxis in antenatal care in Cape Town, South Africa (August, 2019, to October, 2021)

	All women (n=1195)

**Sociodemographic characteristics**	
Age group	
16–24 years	457 (38·2%)
≥25 years	738 (61·8%)
Maternal age, years	26 (23–31)
Gravidity	
Primigravida	405 (33·9%)
Multigravida	790 (66·1%)
Education level completed	
Primary or some primary	581 (48·6%)
Secondary or tertiary	614 (51·4%)
Relationship with current partner	
Married or cohabiting	450 (37·7%)
Not married or not cohabiting	745 (62·3%)
Socioeconomic status	
Low	381 (31·9%)
Moderate or high	814 (68·1%)
**Clinical characteristics**	
Gestational age group at booking	
≤20 weeks	560 (46·9%)
>20 weeks	635 (53·1%)
Gestational age at booking, weeks	21 (15–31)
Sexually transmitted infection diagnosed at baseline (Chlamydia trachomatis, Neisseria gonorrhea, or Trichomonas vaginalis)	
Negative	822 (68·8%)
Positive	373 (31·2%)
**Behavioural characteristics**	
Sexually active (past 3 months)	
Not sexually active	33 (2·8%)
1–4 times per month	686 (57·4%)
5–20 times per month	391 (32·7%)
>20 times per month	85 (7·1%)
Condom use (past 3 months)	
Sometimes or always	298 (24·9%)
Never or rarely	864 (72·3%)
No sex partner	33 (2·8%)
Number of sex partners (past 3 months)	
No sex partner	33 (2·8%)
1	1126 (94·2%)
≥2	36 (3·0%)
Intimate partner violence (past 12 months)	147 (12·3%)
Alcohol use (past 12 months)	
Never	602 (50·4%)
Monthly or less	527 (44·1%)
Weekly or daily	66 (5·5%)
Partner's serostatus	
Concordant HIV-negative	849/1181 (71·9%)
Serodiscordant	20/1181 (1·7%)
Unknown	312/1181 (26·4%)
HIV risk perception at enrolment	
No chance at all	650 (54·4%)
Some or high chance	545 (45·6%)
Depression threshold (Edinburgh Postnatal Depression Scale^20^)	
Above threshold ≥11	89 (7·4%)
Intended pregnancy	398 (33·3%)
Prep initiation at baseline	1009 (84·4%)

Data are median (IQR), n (%), or n/N (%).

**Table 2:** Characteristics of women who initiated PrEP and incidence of first PrEP discontinuation (did not receive repeat PrEP prescription in >3 months) up to 12-months postpartum (a total of 18 months of follow-up)

	Continuation probability at midpoint of 18 months of follow-up on PrEP (95% CI)	Median survival continuation, months (95% CI)	p value	Hazard ratio of discontinuation (95% CI)	Adjusted hazard ratio of discontinuation (95% CI)*

Maternal age, years†	..	..	..	0·99 (0·98–1·00)	0·99 (0·98–1·00)
Gravidity					
Primigravida	0·21 (0·17–0·26)	3 (3–6)	0·047	1 (ref)	1 (ref)
Multigravida	0·26 (0·22–0·29)	6 (3–6)	0·047	0·87 (0·79–0·99)	1·01 (0·86–1·18)
Gestational age at baseline, weeks‡	..	..	..	1·02 (1·01–1·03)	1·02 (1·01–1·02)
Highest educational level§					
Primary or some primary	0·27 (0·23–0·31)	6 (3–6)	..	1 (ref)	1 (ref)
Secondary or tertiary	0·21 (0·18–0·25)	3 (3–6)	..	1·11 (0·99–1·26)	1·10 (0·97–1·25)
Sexually transmitted infection diagnosed at enrolment (Chlamydia trachomatis, Neisseria gonorrhoeae, or Trichomonas vaginalis)					
Negative	0·24 (0·21–0·28)	6 (3–6)	0·86	1 (ref)	1 (ref)
Positive	0·23 (0·19–0·28)	6 (3–6)	0·86	1·02 (0·89–1·16)	1·00 (0·87–1·14)
Intended pregnancy	0·28 (0·24–0·34)	6 (3–6)	0·0060	0·83 (0·73–0·95)	0·90 (0·79–1·14)
Relationship status since latest visit					
Not married or not cohabiting	0·26 (0·23–0·30)	6 (6–6)	0·011	1 (ref)	1 (ref)
Married or cohabiting	0·21 (0·18–0·25)	3 (3–3)	0·011	1·22 (1·08–1·38)	1·32 (1·16–1·50)
Sexually active since last visit					
Not sexually active	0·33 (0·27–0·41)	9 (6–9)	0·0011	1 (ref)	1 (ref)
Sexually active	0·22 (0·19–0·25)	3 (3–3)	0·0011	1·41 (1·20–1·66)	1·47 (1·25–1·74)
Condom use since last visit¶					
Sometimes or always	0·29 (0·24–0·34)	4 (3–6)	<0·0001	1 (ref)	1 (ref)
Never or rarely	0·19 (0·16–0·22)	3 (3–3)	<0·0001	1·37 (1·19–1·59)	1·43 (1·23–1·65)
New partner in past 3 months	0·30 (0·17–0·54)	6 (6–12)	0·18	1·33 (0·90–1·95)	1·26 (0·85–1·86)
Intimate partner violence					
No intimate partner violence experienced	0·25 (0·23–0·28)	6 (6–6)	<0·0001	1 (ref)	1 (ref)
Intimate partner violence experienced	0·05 (0·02–0·15)	3 (3–3)	<0·0001	2·04 (1·59–2·60)	2·03 (1·59–2·59)
Alcohol use in past 3 months					
None	0·22 (0·20–0·25)	6 (3–6)	0·0017	1 (ref)	1 (ref)
Any	0·45 (0·36–0·57)	9 (6–12)	0·0017	0·72 (0·58–0·91)	0·71 (0·56–0·89)
Partner's serostatus since last visit||					
Serodiscordant	0·47 (0·29–0·78)	12 (3–18)	<0·0001	0·58 (0·34–0·99)	0·67 (0·39–1·15)
Unknown	0·01 (0·03–0·14)	3 (3–3)	<0·0001	3·11 (2·51–3·86)	3·03 (2·44–3·77)
Depression reported (EPDS)					
Below threshold (<11)	0·24 (0·22–0·27)	6 (3–6)	0·023	1 (ref)	1 (ref)
Above threshold (≥11)	0·14 (0·06–0·30)	3 (3–6)	0·023	1·58 (1·18–2·12)	1·53 (1·14–2·05)

Data are probability, median, or hazard ratio (95% CI), unless otherwise stated. PrEP=pre-exposure prophylaxis. EPDS=Edinburgh Postnatal Depression Scale.^20^

* Adjusted for maternal age (continuous), gestational age at baseline (continuous), and educational level (categorical).

† Adjusted for gestational age at baseline (continuous) and highest educational level (categorical).

‡ Adjusted for maternal age (continuous) and highest educational level (categorical).

§ Adjusted for maternal age (continuous) and gestational age at baseline (continuous).

¶ Among those sexually active since last visit (n=860).

|| Among those with partners (n=1064).

**Table 3:** HIV incidence rates among all participants in the PrEP-PP cohort study (n=1195), stratified by pregnant and postpartum status, August, 2019, to February, 2023

	Time in study, woman-years	Seroconversions	Incidence rate, per 100 women-years (95% CI)

Total HIV seroconversions	1673·8	16	0·96 (0·49–1·42)
Pregnant	359·8	1	0·28 (0·22–0·33)
Postpartum	1313·9	15	1·14 (0·64–1·88)
≤6 months postpartum	560·9	5	0·89 (0·11–1·67)
6–12 months postpartum	753·0	10	1·33 (0·51–2·15)
Discontinued PrEP	1516·9	14	0·92 (0·49–1·52)
Never on PrEP	156·8	2	1·28 (0·48–3·03)

PrEP=pre-exposure prophylaxis.

## Data Availability

Study de-identified data is available for sharing with a signed data access agreement, which includes the data dictionary, study protocols, statistical analysis plan, and informed consent forms, following this publication and upon request via djosephdavey@mednet.ucla.edu.

## Research in context

### Evidence before this study

We searched ClinicalTrials.gov and PubMed from inception to Oct 15, 2023, using the search terms “PrEP” or “pre-exposure prophylaxis” or “TDF/FTC” or “HIV prevention” and “pregnancy” or “breastfeeding” or “lactating” or “postpartum”. Our search yielded 258 publications and 13 ongoing studies. Clinical trials in the past decade have focused on the safety and effectiveness of oral tenofovir disoproxil fumarate and emtricitabine pre-exposure prophylaxis (PrEP) among pregnant and lactating people (PLP). These data have provided valuable and reassuring insights into the safety and efficacy of PrEP, informing both WHO guidelines and subsequent 2021 South African National Guidelines to include offering PrEP to PLP. In the past 5 years, studies have focused on the implementation of oral PrEP to understand the barriers to and facilitators of PrEP use among PLP. The use of oral PrEP is rising among PLP, with notable successes in Kenya, South Africa, and Zimbabwe. Previous studies have highlighted existing physical and social barriers contributing to suboptimal PrEP use among PLP, such as difficulties in clinical access, burden of a daily pill regimen, side-effects, insufficient interpersonal support, and PrEP-related stigma. Few studies have integrated PrEP into public sector antenatal or postnatal care to understand its effectiveness, the factors associated with continuation, and incidence of HIV during pregnancy and postpartum among women who used PrEP but discontinued.

### Added value of this study

The PrEP in Pregnancy and Postpartum (PrEP-PP) study addresses the gap in unanswered questions around the topics of PrEP initiation, continuation, and correlations of effective PrEP use among PLP without HIV in antenatal and postnatal care. To our knowledge, this study is one of the first studies in South Africa that is integrated into a public sector antenatal clinic setting and contributes to understanding PrEP use and continuation, barriers and facilitators, and HIV incidence among PLP over time to inform appropriate future interventions, improving PrEP uptake and persistence within this population. PrEP initiation was high among pregnant women, but fewer than a fifth continued to 12-months postpartum, indicating that substantial barriers exist for PLP to persist on PrEP. Women who were married or cohabiting with their partner or those who reported intimate partner violence or condomless sex in the past year were more likely to discontinue PrEP, highlighting the need to reach those with other social and HIV-related risk factors with interventions to promote effective use. Furthermore, in a subset of women who continued PrEP at 6 months, fewer than half had measurable concentrations of tenofovir diphosphate in their dried blood spots, and half of the women with measurable concentrations had low concentrations of tenofovir diphosphate (<2 doses per week), identifying PrEP adherence as another area of necessary focus to improve HIV prevention in PLP. HIV incidence was high among postpartum women who discontinued PrEP.

### Implications of all the available evidence

There is an urgent need for PrEP integration into both antenatal and postpartum care. Additionally, the development of alternative, effective interventions to improve PrEP persistence and adherence in this population is crucial, including exploring strategies such as differentiated service delivery of PrEP, incorporating PrEP into settings that better capture postpartum women, and offering long-acting PrEP modalities during pregnancy and the postpartum period.
