# Supplementary material for: Initiation and continued use of oral pre-exposure prophylaxis among pregnant and postpartum women in South Africa (PrEP-PP): a demonstration cohort study
Source: Lancet HIV. Author manuscript; Available in PMC 2026 Aug 6. (PMC13444643; doi:10.1016/S2352-3018(24)00240-6)
Supplement: Supplementary file 1 [file NIHMS2183467-supplement-French_abstract.pdf]

THE LANCET
HIV

Supplementary appendix 1

This translation in French was submitted by the authors and we reproduce it as supplied. It has not been peer reviewed. *The Lancet*'s editorial processes have only been applied to the original in English, which should serve as reference for this manuscript.

Cette traduction en français a été proposée par les auteurs et nous l'avons reproduite telle quelle. Elle n'a pas été examinée par des pairs. Les processus éditoriaux du *Lancet* n'ont été appliqués qu'à l'original en anglais et c'est cette version qui doit servir de référence pour ce manuscrit.

Supplement to: Joseph Davey DL, Mvududu R, Mashele N, et al. Initiation and continued use of oral pre-exposure prophylaxis among pregnant and postpartum women in South Africa (PrEP-PP): a demonstration cohort study. *Lancet HIV* 2024; **11:** e746–55.

Contexte: Lorsqu'elle est utilisée efficacement, la prophylaxie pré-exposition orale (PrEP ; fumarate de ténofovir disoproxil et emtricitabine) prévient l'acquisition du VIH par la mère et réduit le risque de transmission verticale. Notre étude visait à mieux comprendre l'initiation, l'utilisation continue et l'observance de la PrEP chez les femmes enceintes et en post-partum.

Méthodes: L'étude PrEP in pregnancy and postpartum (PrEP-PP) est une étude de cohorte de démonstration qui a recruté des femmes enceintes âgées de 16 ans et plus sans VIH participant à leur première visite de soins prénatals au Cap, Afrique du Sud, entre le 29 août 2019 et le 10 octobre 2021. Les femmes éligibles et consentantes ont été suivies tous les trimestres jusqu'à 12 mois après accouchement avec des tests VIH réguliers et une offre de PrEP avec des conseils d'observance continus. Le principal résultat était la répartition des femmes tout au long de la cascade de PrEP (c'est-à-dire l'initiation et la poursuite jusqu'à 12 mois après l'accouchement) avec des rapports de risque (HR) bruts et ajustés. Français Nous rendons également compte de l'incidence du VIH selon la grossesse et le statut post-partum.

Résultats: 1195 femmes enceintes ont été recrutées et suivies (âge médian 26 ans, IQR 23-31 ; âge gestationnel médian 21 semaines, IQR 15-31); 1009 (84.4%) ont commencé la PrEP à l'inscription. Parmi les femmes qui ont commencé la PrEP à l'inscription, 668 (67.5%) sur 990 ont continué la PrEP au suivi à 1 mois, 485 (49.9%) sur 972 ont continué à 3 mois, 392 (39.4%) sur 994 à 6 mois et 275 (27.4%) sur 1005 à 12 mois. Sur 186 femmes qui n'ont pas accepté la PrEP à l'inscription, 70 (37.6%) sur 186 ont ensuite commencé la PrEP. Au total, 200 (18.6%) des 1076 femmes ont continué la PrEP 12 mois après l'accouchement. Sur 186 femmes qui n'ont pas commencé la PrEP au départ, 70 (37.6%) ont ensuite commencé la PrEP au cours de l'étude. Les facteurs associés à l'arrêt de la PrEP jusqu'à 12 mois après l'accouchement comprenaient le fait d'être marié ou de cohabiter (HR ajusté 1.32, IC à 95% 1.16–1.50), les rapports sexuels sans préservatif depuis la dernière visite (1.43, 1.23–1.65), le fait de signaler des violences conjugales (2,03, 1,59–2,59) ou la dépression au cours des 12 derniers mois (1,53, 1,14–2,05). Au total, 16 femmes ont subi une séroconversion sur une période de 1673·8 années-femmes (taux d'incidence du VIH de 0·96 pour 100 années-femmes, IC à 95% 0·49–1·42); 14 ont arrêté la PrEP et deux n'ont jamais commencé la PrEP. L'incidence du VIH était de 0·28 pour 100 années-femmes pendant la grossesse (IC à 95% 0·22–0·33), et le taux d'incidence était de 1·77 pour 100 années-femmes (0·53–5·90) 0–6 mois après l'accouchement et de 2·19 pour 100 années-femmes (0·61–7·83) 6–12 mois après l'accouchement par rapport aux femmes enceintes.

Interprétation: Il est urgent d’intégrer la PrEP dans les soins prénatals et postnatals et dans les interventions qui s’attaquent aux obstacles à une utilisation continue, y compris des conseils ciblés pendant la grossesse et le post-partum pour réduire l’arrêt de la PrEP.
