# Supplementary material for: Initiation and continued use of oral pre-exposure prophylaxis among pregnant and postpartum women in South Africa (PrEP-PP): a demonstration cohort study
Source: Lancet HIV. Author manuscript; Available in PMC 2026 Aug 6. (PMC13444643; doi:10.1016/S2352-3018(24)00240-6)

THE LANCET
HIV

Supplementary appendix 2

This appendix formed part of the original submission and has been peer reviewed. We post it as supplied by the authors.

Supplement to: Joseph Davey DL, Mvududu R, Mashele N, et al. Initiation and continued use of oral pre-exposure prophylaxis among pregnant and postpartum women in South Africa (PrEP-PP): a demonstration cohort study. *Lancet HIV* 2024; **11:** e746–55.

Supplementary Table 1. Objective measured tenofovir diphosphate (TFV-DP in dried blood spots (DBS) at 6-month visit (n=186 postpartum women) using odds ratios (crude and adjusted) among participants who had dried blood spots (DBS) taken at six-month follow-up. Multiple logistic regression models for each characteristic of interest.

	**Overall**	**Any TFV present**	**No TFV present**	**OR of no TFV present**	**aOR of no TFV present**[1]
	N (%)	n (%)	n (%)	OR (95% CI)	aOR (95% CI)
**Total participants**	**186**	**86 (46%)**	**100 (54%)**		
SOCIO-DEMOGRAPHIC CHARACTERISTICS					
**Maternal age (median, IQR), years**[2]	27 (23-32)	29 (25-34)	26 (23-30)	**0·94 (0·90, 0·99)**	**0·93 (0·88, 0·98)**
**Education level completed**[3]					
Primary or some primary	95 (51%)	51 (54%)	44 (46%)	REF	REF
Secondary or Tertiary	91 (49%)	35 (38%)	56 (62%)	**1·85 (1·04, 3·34)**	1·81 (0·99, 3·33)
**Relationship Married/cohabiting**					
At enrolment	113 (61%)	51 (45%)	62 (55%)	1·12 (0·62, 2·02)	0·89 (0·46, 1·69)
Since last visit	78 (42%)	34 (44%)	44 (56%)	1·20 (0·67, 2·17)	1·60 (0·84, 3·11)
CLINICAL CHARACTERISTICS					
**GA at booking (median, IQR), weeks**[4]	19 (13-26)	19 (14-26)	19 (12-25)	0·98 (0·95, 1·01)	0·96 (0·93, 1·00)
**Any STI at enrolment (CT/NG/TV)**					
Negative	118 (63%)	52 (44%)	66 (56%)	REF	REF
Positive	68 (37%)	34 (50%)	34 (50%)	0·79 (0·43, 1·43)	0·68 (0·36, 1·28)
BEHAVIORAL CHARACTERISTICS (LAST 3 MONTHS FROM BASELINE COMPARED TO SINCE LAST VISIT)					
**Sexually active since last visit**					
Not sexually active	58 (31%)	27 (47%)	31 (53%)	REF	REF
Sexually active	128 (69%)	59 (46%)	69 (54%)	1·37 (0·72, 2·64)	1·39 (0·70, 2·80)
**Condom use since last visit***					
Sometimes/always	52 (44%)	24 (46%)	28 (54%)	REF	REF
Never/rarely	76 (56%)	35 (46%)	41 (54%)	0·84 (0·42, 1·66)	0·83 (0·40, 1·72)
**New partner in last 3m**	6 (4%)	3 (50%)	3 (50%)	0·86 (0·15, 4·76)	1·00 (0·17, 5·77)
**Pregnancy intention**					
Non-intended pregnancy	123 (66%)	55 (45%)	68 (55%)	REF	REF
Intended pregnancy	63 (34%)	31 (49%)	32 (51%)	0·83 (0·45, 1·54)	0·95 (0·48, 1·86)
**Inter-personal violence (IPV)**					
No IPV experienced	178 (96%)	83 (47%)	95 (53%)	REF	REF
IPV experienced	8 (4%)	3 (38%)	5 (62%)	1·31 (0·36, 5·27)	1·29 (0·21, 3·45)

* Among those sexually active since last visit
[1] Adjusted for maternal age (continuous), gestational age at baseline (continuous) and highest educational level (categorical)
[2] Adjusted for gestational age at baseline (continuous) and highest educational level (categorical)
[3] Adjusted for maternal age (continuous) and gestational age at baseline (continuous)
[4] Adjusted for maternal age (continuous) and highest educational level (categorical)
Pre-exposure prophylaxis, PrEP; N, number of participants; IQR, Interquartile range; GA, gestational age; STI, Sexually transmitted infection; TFV, Tenofovir diphosphate; REF, reference category

Supplementary Table 2: Median pregnancy and postpartum time in weeks at each study visit in PrEP-PP (2018-2023) in Cape Town, South Africa

	Baseline	3-month visit	6-month visit	9-month visit	12-month visit	15-month visit	18-month visit
GA at visit (median, IQR)	21 (15-31)	31 (26-36)	38 (35-40)				
**weeks (min-max)**	**4 - 41**	**8 - 43**	**20 - 42**				
Time postpartum (median, IQR)		9 (7-11)	14 (8-22)	22 (15-32)	35 (28-45)	48 (41-58)	60 (53-70)
**weeks (min-max)**		**4 - 24**	**4 - 37**	**2 - 50**	**18 - 63**	**28 - 70**	**40 - 78**

Supplementary Figure 1. Consort of PrEP-PP cohort follow up in pregnant women, South Africa (2018-2023)

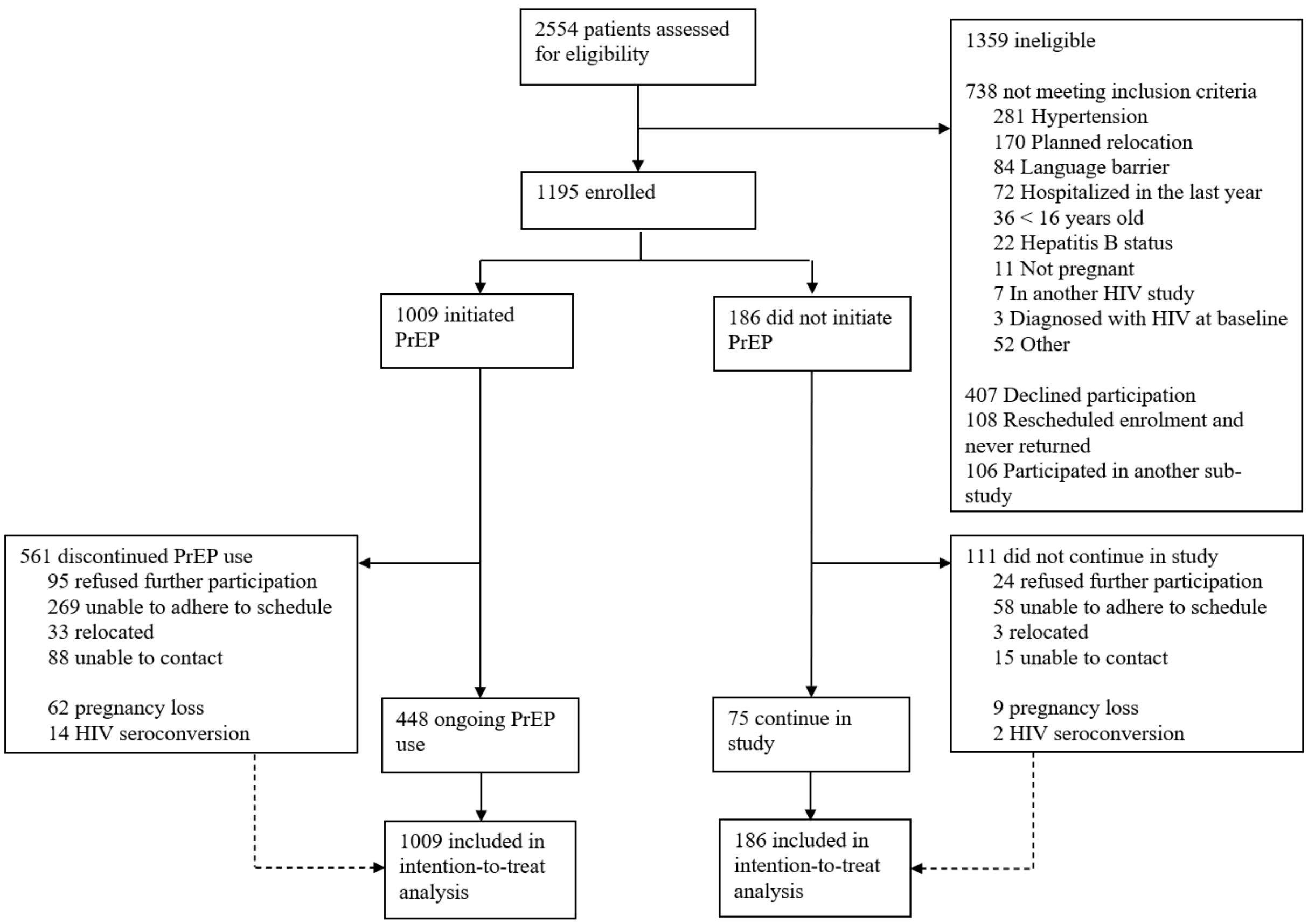

Supplementary Figure 2. Kaplan-Meier curves of first discontinuation of PrEP during (a) pregnancy and (b) 12-months postpartum periods in PrEP-PP study (2018-2023).

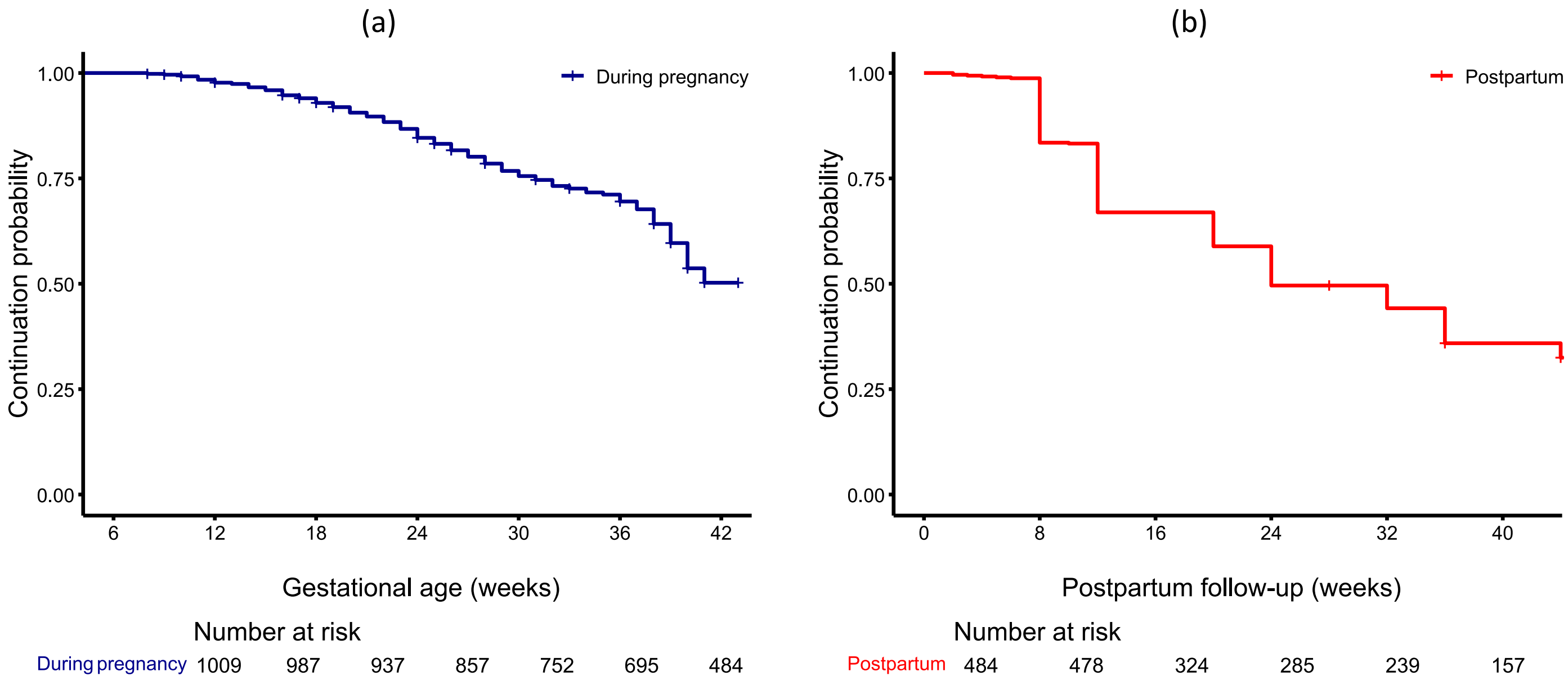

Supplement: Supplementary file 2 [file NIHMS2183467-supplement-Supplemental_tables.pdf]
